# Delayed Lower Motor Neurone Facial Nerve Palsy Following a Traumatic Head Injury

**DOI:** 10.7759/cureus.25753

**Published:** 2022-06-08

**Authors:** Amir Habeeb

**Affiliations:** 1 Otolaryngology, Cambridge University Hospitals, Cambridge, GBR

**Keywords:** rare diagnosis, emergency medicine, trauma, ent surgery, head and neck trauma

## Abstract

Delayed facial nerve weakness secondary to head injury is rare. The mechanism of immediate facial nerve paralysis is obvious, however, the delayed presentation remains disputed. We report a 58-year-old gentleman who presents 6 days after being discharged following head trauma with a 2-day history of facial nerve paralysis (House-Brackmann grade 6). Computed tomography (CT) head showed a minimally displaced longitudinal squamous temporal bone fracture initially with nerve conduction studies and electromyograpy revealing a relative reduction in left facial motor amplitudes with moderate recruitment. He showed good progress following high-dose steroids and conservative management. Early involvement of ear, nose and throat (ENT) surgeons is crucial. The use of both high-resolution CT scanning and nerve conduction studies will help guide management as early as possible and improve outcomes in these patients.

## Introduction

Delayed facial nerve weakness secondary to head injury is rare. Its mechanism is unclear although there are a few postulated pathogeneses. Not only does facial paralysis affect the way the patient looks but it can be associated with a loss of taste, hyperacusis, decreased salivation and tear secretion contributing to dry eye syndrome and if untreated could cause keratopathy and vision loss. Different management options exist and there are discrepancies amongst surgeons as to when to intervene surgically. Facial nerve palsies can be detrimental to a patient’s quality of life and early recognition and better understanding of delayed presentation of facial nerve palsies following trauma can improve outcomes in these patients. The aim of this case report is to highlight important identifying features of delayed facial nerve palsy, its investigation and potential management options.

## Case presentation

**Figure 1:**
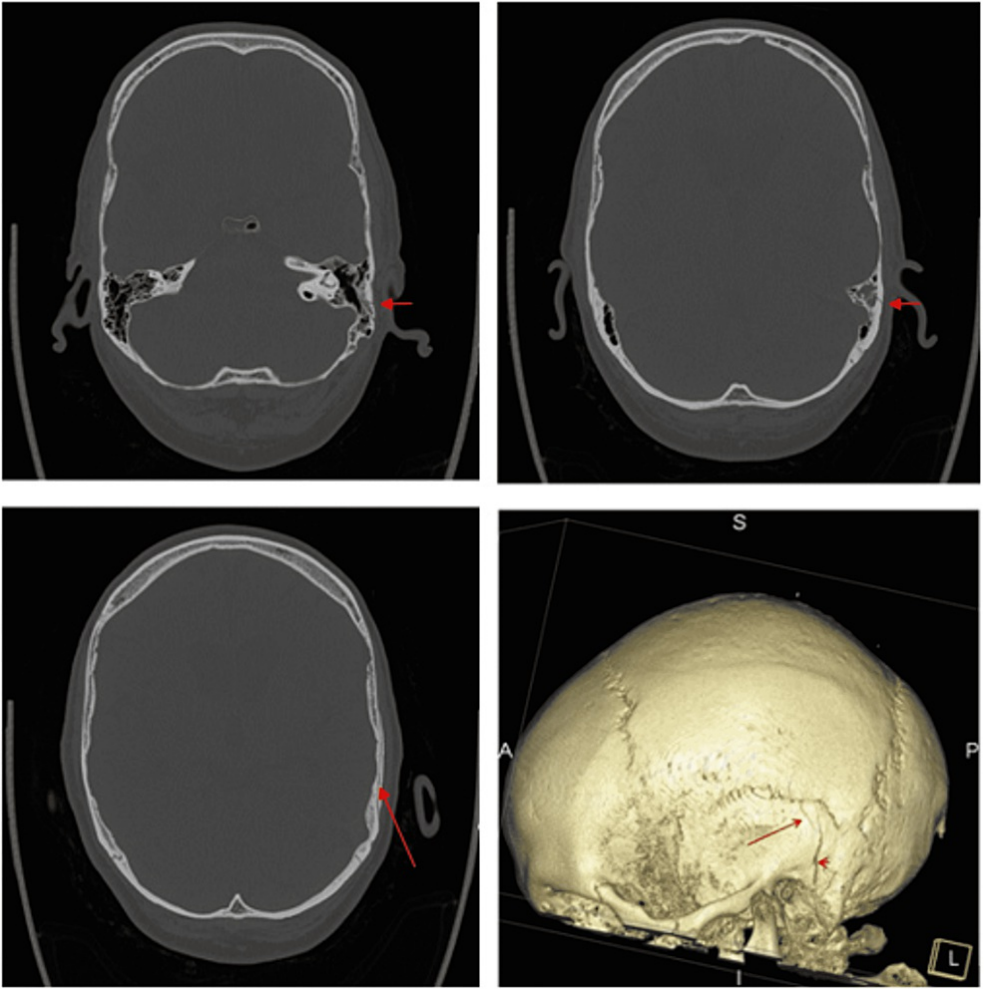
CT head showing undisplaced left mastoid and longitudinal squamous temporal fracture.

**Figure 2:**
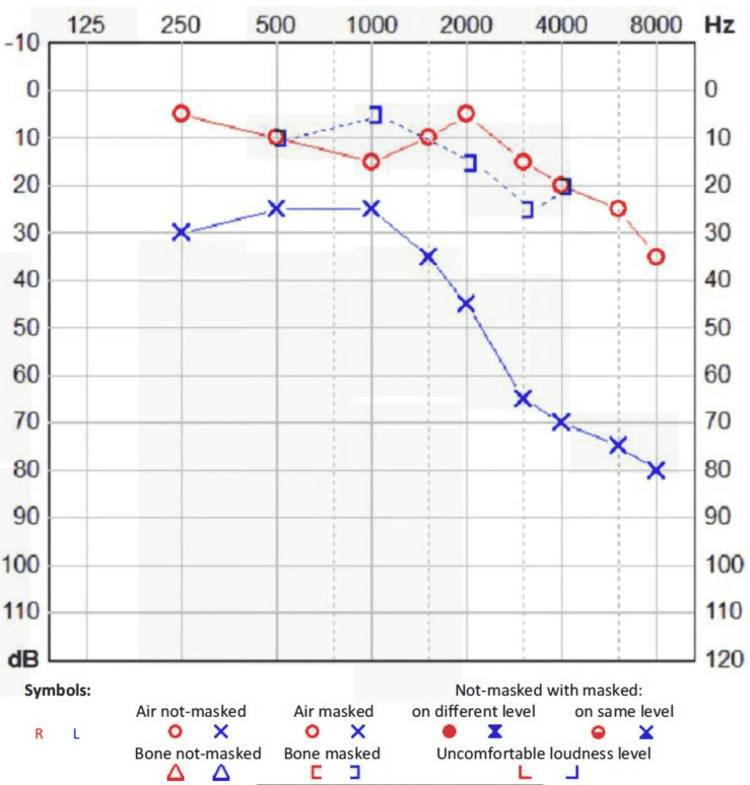
An audiogram showing left-sided conductive hearing loss. There is also mixed to sensorineural hearing loss at higher frequencies.

We present a case of a 58-year-old gentleman with a background of extensive head trauma following a fall from the top of the stairs discharged one week prior with a CT head that showed a minimally displaced longitudinal fracture of the left squamous temporal bone extending into the left mastoid process. Clinically, he had no neurological deficits although he had haemotympanum so was told to come to the ENT clinic for a follow-up. Six days later he presents with a 2-day history of left-sided facial weakness and a painful eye. He also noted one episode of bright red bloody discharge from the left ear. On examination, he had non-forehead sparing facial nerve palsy on the left (House-Brackmann grade 6). Tuning fork tests revealed left-sided conductive hearing loss and otoscopy showed haemotympanum. Early exposure keratopathy was seen in his left eye. CT head on admission revealed new right temporal lobe contusion, with an adjacent shallow subdural haematoma and associated subarachnoid haemorrhagic component as well as a minimally displaced fracture of the left longitudinal squamous temporal bone, extending into the left mastoid process. CT head 10 days later showed no evidence of new intracranial haemorrhage. There was partial residual opacification of left mastoid air cells with an undisplaced left mastoid and longitudinal squamous temporal fracture (Figure 1). An audiogram revealed largely left conductive hearing loss with mixed to sensorineural hearing loss at higher frequencies (Figure 2). Nerve conduction studies revealed a relative reduction in left facial motor amplitudes. On electromyography, moderate recruitment was observed in the left orbicularis oculi and orbicularis oris. In conclusion, findings were in keeping with a left facial nerve palsy of at least moderate severity with a degree of nerve continuity.

60 mg prednisolone was prescribed for 1 week followed by a reducing regimen over 3 weeks. His eye was washed out and drops were given-hypromellose 0.3% drops hourly, chloramphenicol 1% ointment four times daily. In addition to this, his eyelid was closed with tape at night. A referral was made for facial physiotherapy on discharge. Due to the chronology of events and nerve conduction study results, he was deemed unlikely to benefit from surgical intervention so was discharged with conservative management measured for follow up by oculoplastics, ophthalmology, head trauma clinic and ENT in the community within 2-4 weeks of discharge. His facial nerve palsy was seen in the clinic 3 weeks after discharge to be resolved at the time of writing to House-Brackmann 3 with conservative measures.

## Discussion

Being a motor cranial nerve, the facial nerve is most commonly affected in closed head injuries [1]. Immediate facial nerve palsies following trauma arise secondary to severance of the nerve while delayed presentations are likely as a result of bleeding into the facial canal-a space that is only 30-50% occupied by the facial nerve with blood vessels and connective tissue encompassing it [2]. An expanding haematoma can compress the nerve and cause ischaemic damage. The extent of the damage depends on the degree of pressure-mild pressure may cause neuropraxia or conduction block due to segmental demyelination while higher pressures could cause permanent axonal damage with subsequent denervation [3]. Other theories of pathogenesis involve nerve oedema, delayed arterial spasm or compression of the facial nerve by bony fragments [4,5]. It is important to note that delayed facial nerve palsy should not be confused with Bell’s palsy which has very different pathogenesis in that it is idiopathic.

The vast majority of temporal bone fractures are longitudinal while only 10% are transverse, although in transverse fractures 30-50% cause facial paralysis due to direct nerve trauma and are more likely to be immediate while in longitudinal fractures 10-25% will experience facial nerve paralysis and 88% of these will have delayed facial nerve palsies. This is accompanied by passing through the external auditory canal and tearing the tympanic membrane [3].

One of the biggest issues is the initial diagnosis of a facial nerve palsy which may be mild in the early stages and early identification may assist in preventing delayed presentation. In our case, a full clinical examination by ENT specialists following initial head trauma revealed no sign of facial nerve palsy however the patient did have a minimally displaced left longitudinal temporal bone fracture and haemotympanum. According to Puvanendran et al., of the 6304 cases of head injuries studied, there were no patients that had delayed facial palsies without haemotympanum which may indicate that delayed facial palsies may be caused by blood collecting in the middle ear and affecting the facial nerve if the bony facial canal is dehiscent which is a common anatomical variant [3]. In contrast to Briggs and Potter who found that 38.5% of cases with delayed facial weakness had both basal skull fracture reported and haemotympanum present [2]. In essence, having both these features in the first instance increases your chances of having delayed facial weakness. Topodiagnostic mapping in the first instance would negate examination subjectivity, although there can be a delay in requesting these studies and immediate palsy from the severance of the facial nerve would have given rise to an instant high-grade palsy that should not be missed by an ENT specialist.

CT head evaluation for facial nerve trauma secondary to basal skull fracture is usually inadequate for assessment of the temporal bone but rather dedicated high-resolution 1-mm CT sections should be performed for a higher chance of visualising facial nerve compromise [6]. Although facial nerve functional lesions of branches could potentially be identified by the Schirmer test, stapedius reflex or gustatory reflex, this is subjective and only reliable in patients with total paralysis [7]. In our case, high-resolution CT was not performed at any point throughout the case, although neuroradiologists were able to comment on facial canal integrity and given the nerve conduction study results, ENT surgeons opted to reduce radiation exposure by avoiding rescanning. Nerve conduction studies as well as electromyography can help identify nerve recruitment status providing insight as to whether the patient should be managed conservatively [8].

Regarding intervention options, conservative treatment with prednisolone 1 mg/kg for 1 week followed by a reducing regimen is usually enough while surgery and reanimation are only indicated where electromyography shows 90% nerve denervation within 6 days after palsy onset [9]. One-year follow-up by Li et al. revealed 81.8% fully recovering with conservative management only. In our case, an improvement from House-Brackmann 6 to 3 was observed in roughly a 6-week timescale using conservative measures and, although documentation using this scale can be subjective, there was an improvement nonetheless. They were also able to show a male predisposition of 4:1 and 88.6% of palsies occurring within 2 weeks of initial head trauma which both agree with our case [9]. Interestingly, Li et al. showed that 28.6% of cases had simple brain contusions or subdural haematomas without a temporal bone fracture suggesting that high levels of shock injury can suffice in causing delayed facial nerve paralysis [9]. Although amongst ENT surgeons in the UK, conservative management is the preferred choice, where surgical management is being considered, the appropriate time of decompression remains a subject of controversy [10]. The type of approach will depend on the degree of the facial nerve lesion. In most cases, this extends up to the geniculate ganglion meaning a translabyrinthine and middle cranial fossa approach is required to decompress the entire facial nerve. If the geniculate ganglion is not impacted then a mastoid approach can be used [10]. There is a school of thought that early facial nerve decompression allows early expansion of the nerve, relieving any oedema but also can remove bony spicules that project onto the nerve and drain the blood collecting in the fallopian canal. The more this is delayed, the heightened the chances of fibrotic bands forming after repairing as well as bony spicules permanently damaging the facial nerve. In addition, a longer delay may result in more ischaemic time which leads to degeneration of the facial nerve [11,12]. The operation does not come without its negatives-facial nerve function will likely be delayed as long as even 12 months after surgical decompression despite some reports of immediate resolution [13]. Potential complications include cerebrospinal fluid leak using the middle cranial fossa approach as well as other risks of seizure, stroke, haematoma and meningitis, although these are rare [14].

## Conclusions

To summarise, delayed facial nerve palsy pathogenesis is likely related to bleeding into the facial canal as a result of vessel trauma, bony spicules, nerve oedema or delayed arterial spasm. Having both temporal bone fracture and haemotympanum increases your chances of having delayed facial nerve palsy by a considerable degree. Emergency medicine doctors are key in taking a detailed history, performing a thorough examination of specifically the facial nerve and involving ENT surgeons early on. Dedicated high-resolution 1-mm CT sections should be performed to adequately visualise the facial nerve following head trauma and surgeons should not rely on functional lesion examination skills in the absence of complete paralysis. Nerve conduction studies and electromyography should be encouraged in the first instance to negate examination subjectivity in detecting facial nerve palsies. Surgical intervention is not usually required and most make full recovery with conservative measurements following 1 week of steroids and a tapering regimen. Early recognition of facial nerve paralysis following head trauma or identification of at-risk individuals will optimise outcomes for these patients as delaying surgical decompression in patients with denervation is associated with worse results.
